# Supplementary material for: Soliton walls paired by polar surface interactions in a ferroelectric nematic liquid crystal
Source: Nat Commun. 2022 Jul 7;13:3932. doi: 10.1038/s41467-022-31593-w (PMC9262936; doi:10.1038/s41467-022-31593-w)
Supplement: Supplementary file 1 — Supplementary Information [file 41467_2022_31593_MOESM1_ESM.pdf]

SUPPLEMENTARY INFORMATION

Soliton walls paired by polar surface interactions in a ferroelectric nematic liquid crystals

Bijaya Basnet[1,2,§], Mojtaba Rajabi[1,3,§], Hao Wang[1,§], Priyanka Kumari[1,2], Kamal Thapa[1,3], Sanjoy Paul[1], Maxim O. Lavrentovich[4], and Oleg D. Lavrentovich[1,2,3]*

Affiliations:

[1]*Advanced Materials and Liquid Crystal Institute, Kent State University, Kent, OH 44242, USA*

[2]*Materials Science Graduate Program, Kent State University, Kent, OH 44242, USA*

[3]*Department of Physics, Kent State University, Kent, OH 44242, USA*

[4]*Department of Physics and Astronomy, University of Tennessee, Knoxville, 37996 TN, USA*

[§]These authors contributed equally to the work.

Corresponding author:

*Author for correspondence: e-mail: olavrent@kent.edu, tel.: +1-330-672-4844.

Keywords: ferroelectric nematic liquid crystal, domain walls, polar surface anchoring, soliton-soliton pairs

I. Synthesis of DIO

1. Materials and Methods

All reagents and solvents were available commercially and used as received unless otherwise stated. ^{1}H (400 MHz), and ^{13}C (100 MHz) spectra were recorded on a Bruker NMR spectrometer using $CDCl_3$ as solvent. Chemical shifts are in δ unit (ppm) with the residual solvent peak as the internal standard. The coupling constant (J) is reported in Hertz (Hz). NMR splitting patterns are designed as follows: s, singlet; d, doublet; t, triplet; and m, multiplet. Column chromatography was carried out on silica gel (230-400 mesh). Analytical thin-layer chromatography (TLC) was performed on commercially coated 60 mesh F_{254} glass plates. Spots on the TLC plates were rendered visible by exposure to UV light. Mass spectra were obtained using a high-resolution instrument Thermo Exactive Plus at the Department of Chemistry, Kent State University.

2. Synthesis

2.1 Synthesis of intermediate **3**.

2,6-Difluoro-4-formaylbenzoic acid (**1**, 1.86 g, 3.65 mmol) and 3-Fluoro-4-(3,4,5-trifluorophenyl)phenol (**2**, 2.42 g, 3.65 mmol) were added into a 100 mL flask, Dicyclohexylcarbodiimide (DCC, 1.92 g, 4.0 mmol) and 4-Dimethylaminopyridine (DMAP, 0.147 g, 0.18 mmol) were added, followed by an addition of 50 mL Dry Dichloromethane (DCM). The mixture was magnetically stirred for 48 hours. Water was added to dissolve DCU and separate the organic and aqueous layers. The organic layer was dried by adding Na_2SO_4, filtered, after which the solvent was evaporated. The crude product was purified by silica gel chromatography with an eluent of hexane/DCM: 1/1 by volume ratio, giving an intermediate **3** as **a** white solid of 1.15 g, with a yield 77 %.

^{1}H NMR (400 MHz, $CDCl_3$) δ ppm: 10.02 (s, 1H), 7.59-7.55 (m, 2H), 7.46 (t, J = 8.63 Hz, 1H), 7.21-7.17 (m, 4H). ^{13}C NMR (100 MHz, $CDCl_3$) δ ppm: 188.44, 162.48, 160.60, 159.88, 158.50, 158.10, 152.45, 150.63, 149.96, 140.84, 140.28, 138.32, 130.84, 124.76, 117.84, 114.78, 113.31, 113.12, 112.95, 112.69, 110.63, 110.37.

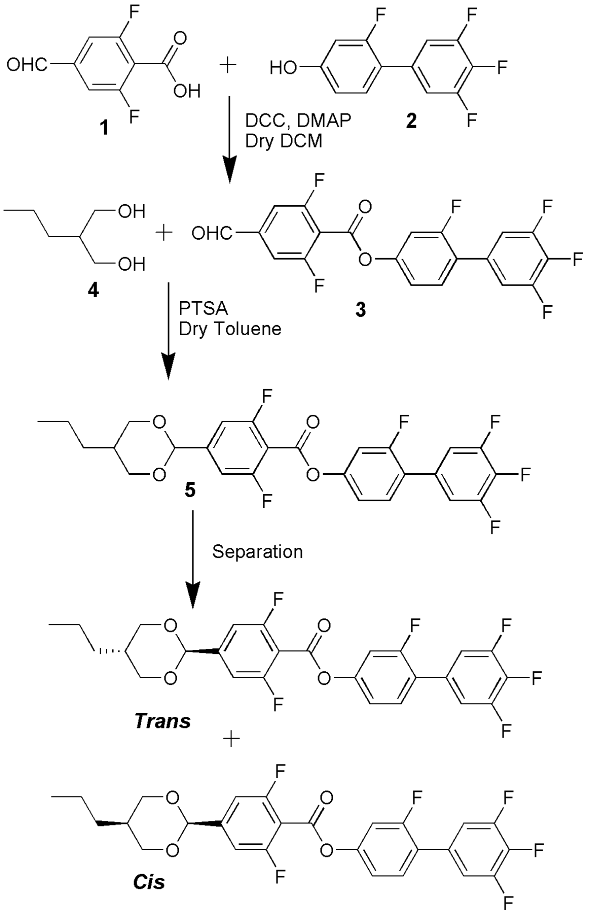

Supplementary Figure 1. Synthetic route of DIO and chemical structures of DIO (*trans*) and its isomer (*cis*).

2.2 Synthesis of target compound **5** (DIO).

The intermediate **3** (0.73 g, 1.78 mmol) and 2-Propyipropane-1,3-diol (compound **4**, 0.23 g, 1.96 mmol) were added into 100 mL flask, *p*-Toluenesulfonic acid monohydrate (PTSA·H_2O, 0.15 g, 0.79 mmol), after which a dry Toluene was added. The mixture was refluxed for 48 hours until work up. After evaporating the solvent, the crude solid was purified through a silica gel column with an eluent of hexane/EA: 10/1 to give two compounds. It turned out that the first spot is the target compound with *trans*-2,6-dioxane structure (0.32 g, yield 35%), and the second spot is the DIO isomer with *cis*-2,6-dioxane structure, as shown in Figure S1. The two isomers showed different peaks of the1,3-dioxane part in the NMR spectra.

DIO: ^{1}H NMR (400 MHz, $CDCl_3$) δ ppm: 7.43 (m, 1H), 7.20-7.14 (m, 6H), 5.40 (s, 1H), 4.27-4.23 (m, 2H), 3.54 (t, J = 11.47 Hz, 2H), 2.14 (m, 1H), 1.34 (m, 2H), 1.10 (m, 2H), 0.93 (t, J = 7.32 Hz, 3H); ^{13}C NMR (100 MHz, $CDCl_3$) δ ppm: 162.18, 160.54, 159.61, 159.24, 158.05, 152.39, 150.96, 149.90, 145.62, 140.73, 138.21, 130.81, 130.63, 124.29, 118.12, 113.29, 113.07, 110.72, 110.46, 110.38, 110.15, 109.34, 98.73, 72.53, 33.85, 30.18, 19.49, 14.15. HR-MS (ESI) calcd. $[C_{26}H_{21}F_6O_4]^+$: 511.1344; found: 511.1339.

DIO isomer:

^{1}H NMR (400 MHz, $CDCl_3$) δ ppm: 7.43 (m, 1H), 7.20-7.14 (m, 6H), 5.50 (s, 1H), 4.13-4.06 (m, 4H), 1.73 (m, 2H), 1.50 (m, 1H), 1.41 (m, 2H), 0.96 (t, J = 7.32 Hz, 3H); ^{13}C NMR (100 MHz, $CDCl_3$) δ ppm: 162.25, 160.57, 159.68, 159.28, 158.08, 152.46, 150.98, 149.98, 145.80, 140.76, 138.24, 130.65, 124.34, 118.14, 113.32, 113.10, 110.49, 110.37, 110.14, 110.11, 109.39, 99.06, 70.66, 33.90, 31.51, 20.51, 14.06. HR-MS (ESI) calcd. $[C_{26}H_{21}F_6O_4]^+$: 511.1344; found: 511.1339.

3. NMR spectra

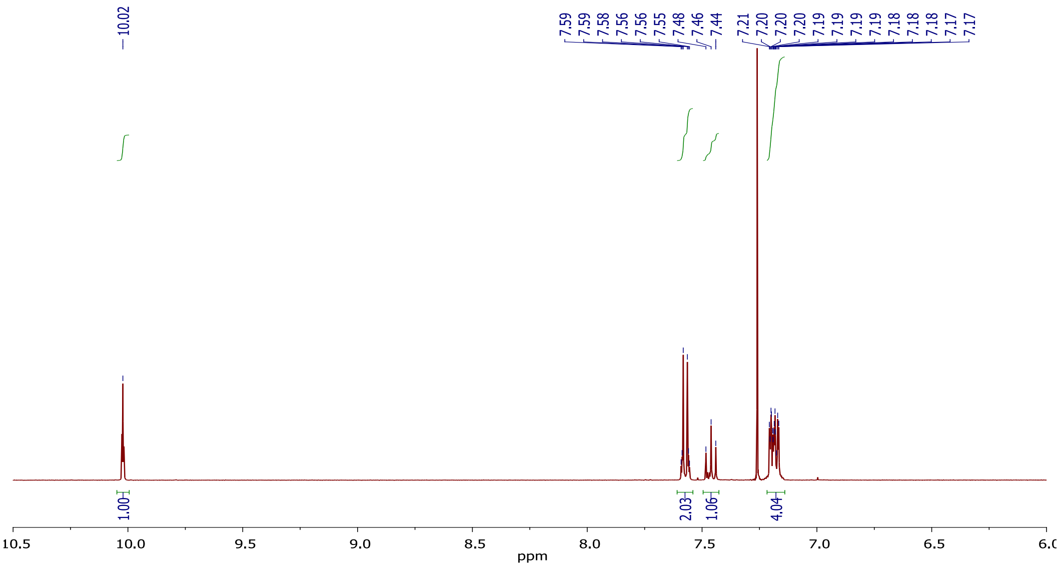

Supplementary Figure 2. ^{1}H NMR (400 MHz, $CDCl_3$) spectrum of intermediate 3.

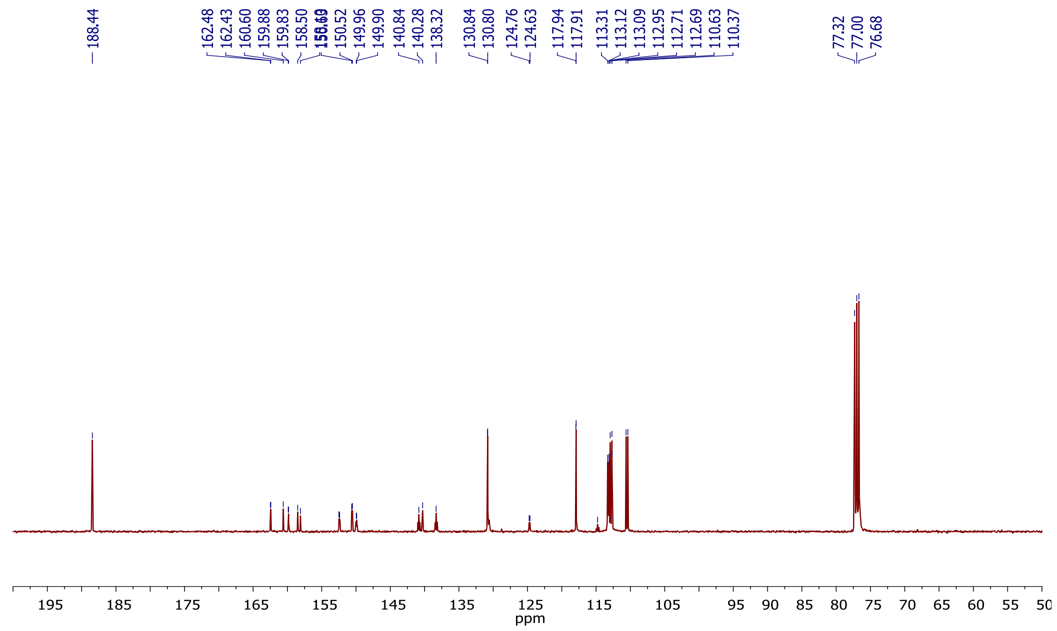

Supplementary Figure 3. ^{13}**C NMR (100 MHz,** $CDCl_3$**) spectrum of intermediate 3.**

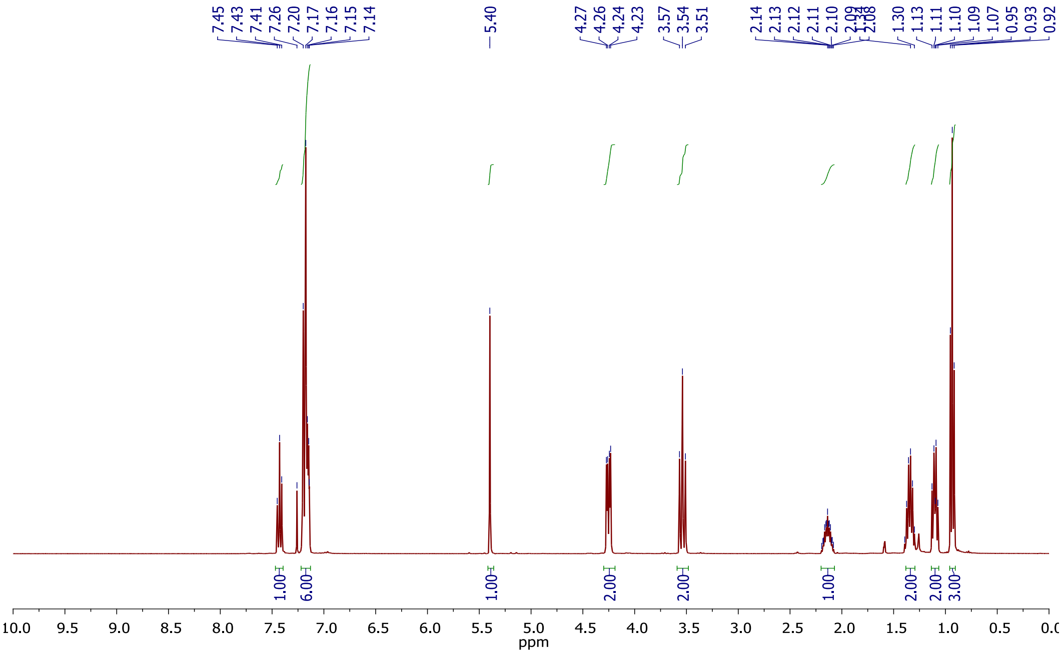

Supplementary Figure 4. ^{1}**H NMR (400 MHz,** $CDCl_3$**) spectrum of DIO.**

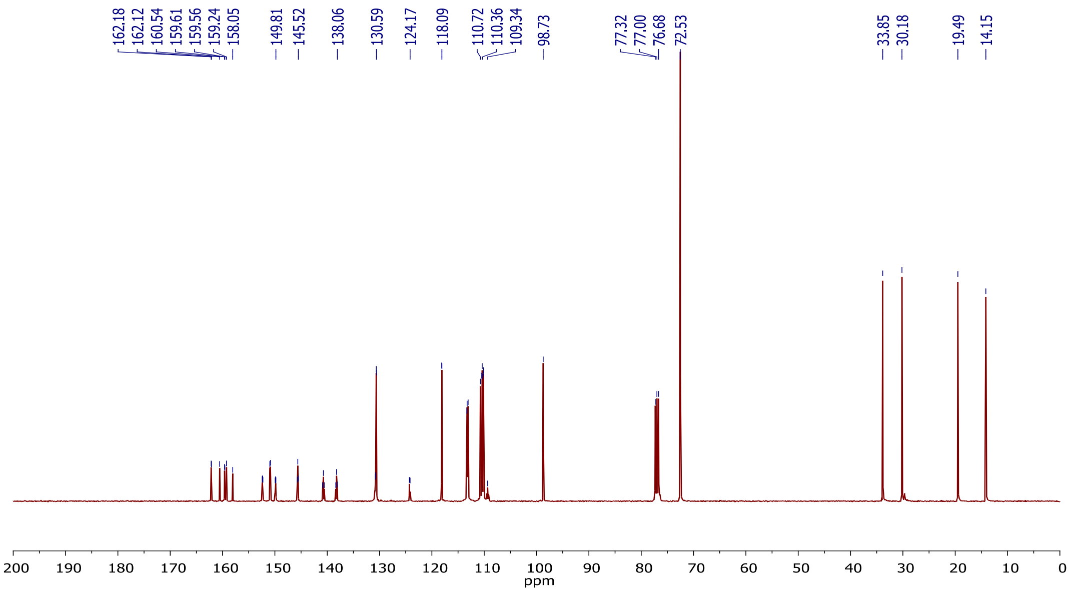

Supplementary Figure 5. ^{13}**C NMR (100 MHz,** $CDCl_3$**) spectrum of DIO.**

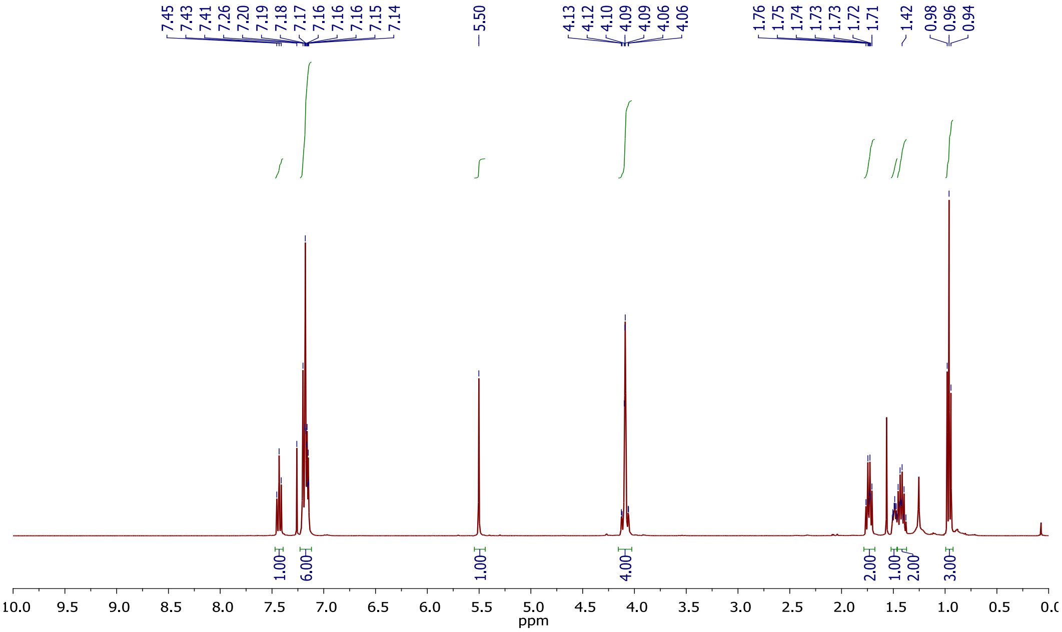

Supplementary Figure 6. ^{1}**H NMR (400 MHz,** $CDCl_3$**) spectrum of DIO isomer.**

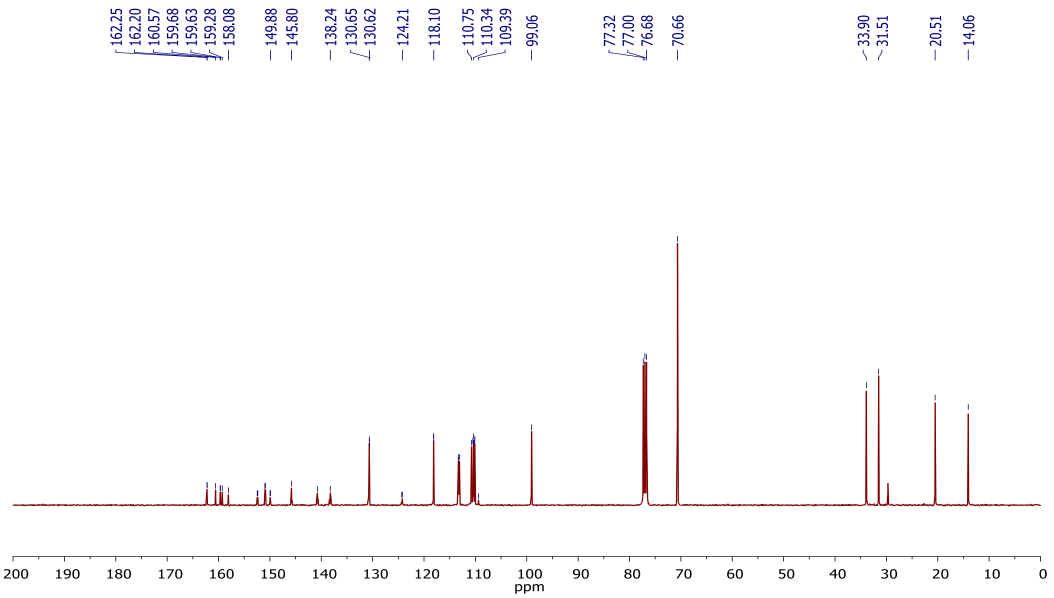

Supplementary Figure 7. ^{13}C NMR (100 MHz, $CDCl_3$) spectrum of DIO isomer.

II. Birefringence of DIO

Birefringence $\Delta n = \Gamma/d$ of DIO was determined by measuring optical phase retardance Γ of planar cells, $d = 6.8$ µm, by PolScope MicroImager (Hinds Instruments). The cell thickness was determined by the interferometric technique. The temperature dependence of Δn measured at λ=535 nm is shown in Supplementary Fig. 8. At 47 ^{o}C, $\Delta n = 0.205$ at 475 nm; 0.201 at 535 nm, and 0.187 at 655 nm. The Cauchy fitting of dispersion yields $\Delta n = a + \frac{b}{\lambda^2}$, where a = 0.1675 and b = 8793 nm^2. Thus at 47 ^{o}C, the birefringence is 0.204 at the transmission wavelength of the blue filter (λ=488 nm); 0.199 for the green filter (λ=532 nm), and 0.189 for the red filter (λ=632.8 nm). For the POM observations with the red filter, $\frac{\pi \Delta n d}{2\lambda}$ =1.02 π, close to the extinction value, since $\sin^2 \frac{\pi \Delta n d}{2\lambda}$=0.002.

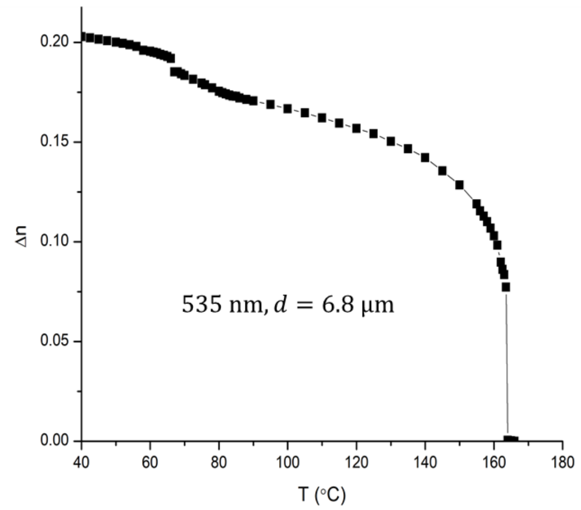

Supplementary Figure 8. Temperature dependence of DIO birefringence; λ=535 nm, cell thickness $d = 6.8$ μm.

III. Width of the domain

The width L_π of the domains that corresponds to the distance between locations with $\varphi = \pi/2$ and $\varphi = 3\pi/2$, shows a weak dependence on the cell thickness d of planar cells. Supplementary Fig. 9 shows the dimensionless ratio L_π/d vs. d.

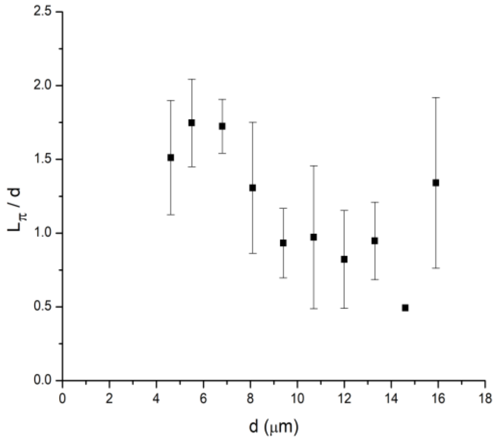

Supplementary Figure 9. The width parameter L_π/d vs. cell thickness d for 360° DW pairs in planar cells. For each d, the data represent an average over the observed DW pairs of total number 64. The error bars represent standard deviation.

IV. Soliton-soliton solution of Euler-Lagrange equation (3).

The solution satisfying the Euler-Lagrange equation (3) with the boundary conditions $\frac{\partial\varphi}{\partial x}(\pm\infty)=0$, $\varphi(\pm\infty)=0$ could also be written in a compact form:

$$\varphi_{\pi\pi}(x)=\pm 2\arctan\left[\sqrt{1+\frac{1}{\omega}}\,\operatorname{csch}\left(\frac{x}{\xi_{\pi\pi}}\right)\right], \quad \text{(S1)}$$

where $\xi_{\pi\pi}=\xi\sqrt{\frac{1}{1+\omega}}$ and $\operatorname{csch}\alpha\equiv 1/\sinh\alpha$. The polarization fields of these W-type pairs of DWs, corresponding to different values of $\omega=W_P/W_Q$ are shown in Supplementary Fig.10a.

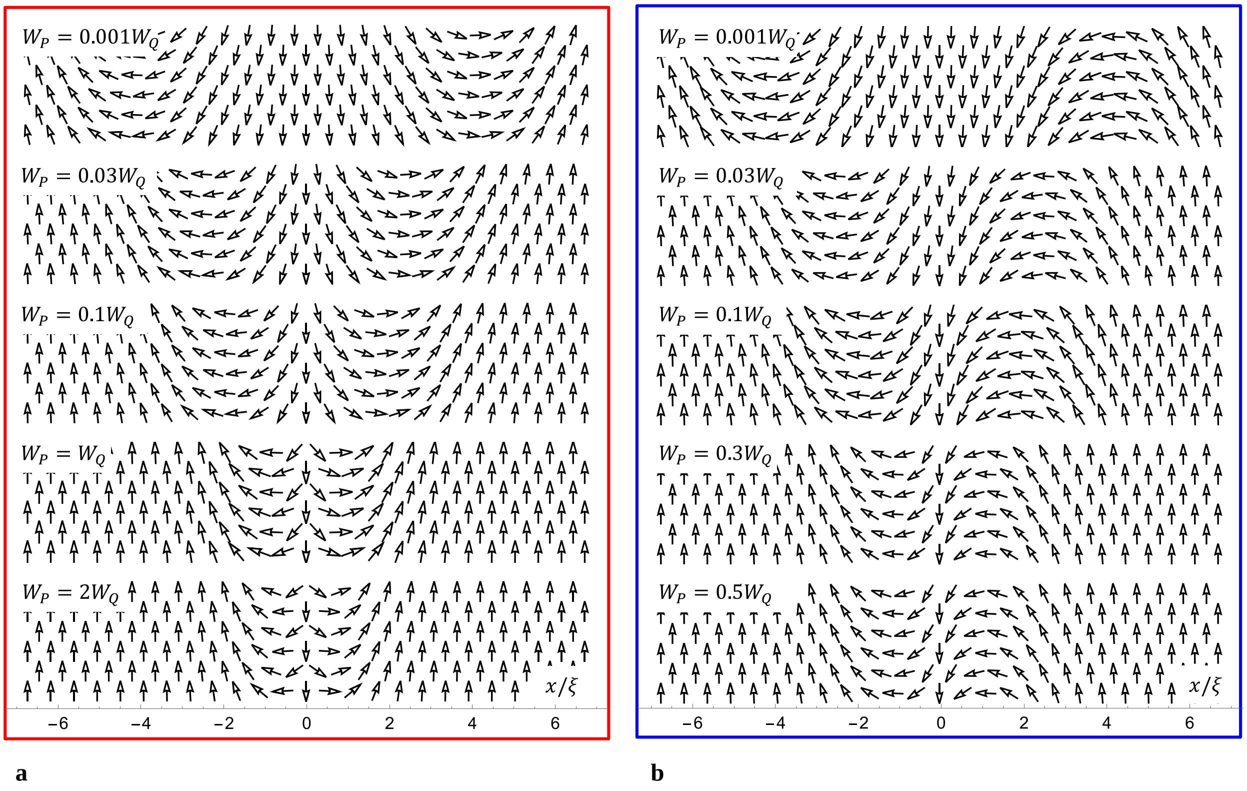

Supplementary Figure 10. Polarization fields in: a, soliton-soliton $\pi\pi$ pairs, Eqs. (7) and (S1) and **b,** soliton-antisoliton $\pi\bar{\pi}$ pairs, Eq. (S2). The solutions, satisfying boundary conditions $\frac{\partial\varphi}{\partial x}(\pm\infty)=0$, $\varphi(\pm\infty)=0$ are shown for different ratios W_P/W_Q. Planar solutions, one constant approximation for bend and splay.

V. Soliton-antisoliton solution of Euler-Lagrange equation (3).

Equation (3) admits a solution for a topologically unprotected soliton-antisoliton $\pi\bar{\pi}$ (or $\bar{\pi}\pi$) pair, satisfying the boundary conditions $\frac{\partial\varphi}{\partial x}(\pm\infty) = 0$, $\varphi(\pm\infty) = 0$,

$$\varphi_{\pi\bar{\pi}}(x) = \mp 2\arctan\sqrt{\frac{2(1+\omega)}{\omega\left[\cosh\left(\frac{2x\sqrt{1+\omega}}{\xi}\right)-1\right]}} = \mp\arccos\frac{\omega\cosh\left(\frac{2x\sqrt{1+\omega}}{\xi}\right)-3\omega-2}{\omega\cosh\left(\frac{2x\sqrt{1+\omega}}{\xi}\right)+\omega+2}, \qquad \text{(S2)}$$

The bar over π implies that the two rotations of polarization are of opposite signs, Supplementary Fig. 10b. These solutions correspond to the experimentally observed S-configurations of the DW pairs, shown in Fig.3a,f.

Supplementary Fig. 11a shows the detailed profile of the azimuthal direction of polarization in the $\pi\bar{\pi}$ pairs for different polar azimuthal anchoring coefficients. Comparison of the S- ($\pi\bar{\pi}$ pairs) and W-configurations ($\pi\pi$ pairs), Supplementary Fig. 11b, shows that the characteristic lengths $L_{\pi/2}$, L_{π}, and $L_{3\pi/2}$ of the two configurations are the same. In order to distinguish the W and S configurations, one needs to use an optical compensator in the POM observations, which helps to elucidate the sense of the director rotation in two neighboring π DWs.

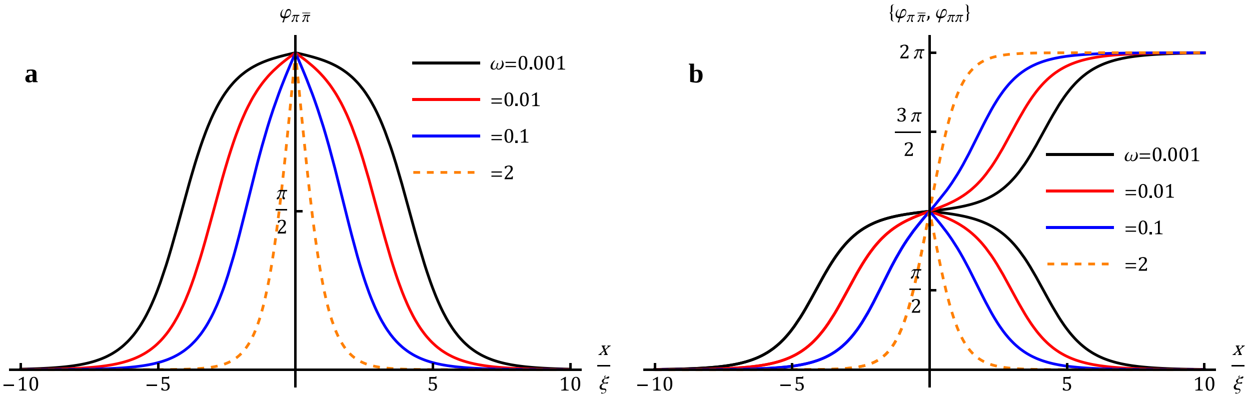

Supplementary Figure 11. Director profiles of planar DW pairs in one-constant approximation: a, soliton-antisoliton $\pi\bar{\pi}$ pairs, Eq. (S2); **b**, comparison of the polarization field in $\pi\pi$ pairs, Eqs. (7) and (S1) and $\pi\bar{\pi}$ pairs, Eq. (S2). The profiles satisfy boundary conditions $\frac{\partial\varphi}{\partial x}(\pm\infty) = 0$, $\varphi(\pm\infty) = 0$ and are plotted for different ratios W_P/W_Q. Note the symmetry of the W- and S-configurations.

VI. Soliton-antisoliton solution of Euler-Lagrange equation (3) enclosing a stable polarization direction.

Equation (3) also admits topologically unprotected soliton-antisoliton $\pi\bar{\pi}$ (or $\bar{\pi}\pi$) solutions, satisfying the boundary conditions $\frac{\partial\varphi}{\partial x}(\pm\infty) = 0$, $\varphi(\pm\infty) = \pi$ and valid when $W_P < W_Q$,

$$\varphi_{\pi\bar{\pi}}(x) = \varphi_\pi\left(\frac{x}{\xi_{\pi\bar{\pi}}} - \frac{\delta_{\pi\bar{\pi}}}{2}\right) + \varphi_\pi\left(-\frac{x}{\xi_{\pi\bar{\pi}}} - \frac{\delta_{\pi\bar{\pi}}}{2}\right) = 2\arctan\left[\sqrt{\frac{\omega}{1-\omega}}\cosh\left(\frac{x}{\xi_{\pi\bar{\pi}}}\right)\right], \quad \text{(S3)}$$

where $\xi_{\pi\bar{\pi}} = \xi\sqrt{\frac{1}{1-\omega}}$, $\delta_{\pi\bar{\pi}} = 2\,\mathrm{arccosh}\sqrt{\frac{1}{\omega}}$. In these configurations, the narrow band represents a stable orientation of polarization, **P** ↑↓ **R** , antiparallel to the rubbing direction.

When $\omega \ll 1$, the walls are well separated, $\Delta x \approx \sqrt{\frac{Kd}{2W_Q}}\ln\frac{4}{\omega}$, and their width $\xi_{\pi\bar{\pi}} \approx \xi\left(1+\frac{\omega}{2}\right)$ approaches ξ. The energy $F_{\pi\bar{\pi}} = 2F_\pi\left[\sqrt{1-\omega} - \omega\,\mathrm{arctanh}\sqrt{1-\omega}\right]$ of the pair is close to the sum of the energies of two π solitons, $F_{\pi\bar{\pi}} \approx 2F_\pi\left[1-\frac{\omega}{2}\left(1+\ln\frac{4}{\omega}\right)\right]$. At $\omega \to 1$, the walls are close, $\Delta x \approx \frac{2\xi}{15}(23-11\omega)$, and their energy vanishes, $F_{\pi\bar{\pi}} \approx \frac{8}{3}\sqrt{2KdW_Q}(1-\omega)^{3/2}$.

It is also interesting to study the soliton with $\phi \to \pi$ as $x \to \pm\infty$ accounting for the difference in the elastic constants and the polar tilt of the director. In the absence of tilt ($\theta = 0$), the soliton will decay to a constant $\phi = \pi$. However, introducing a tilt allows for the azimuthal angle ϕ to transition to $\phi = 0, 2\pi$ where the anchoring is favorable. In this case, the soliton breaks up into two π-solitons which move apart from each other, relaxing the system into a uniform $\phi = 0, 2\pi$. The motion of these walls for $\kappa = K_1/K_3 = 10$, $d/\xi_3 = 20$, $\omega = 0.1$, $K_2/K_3 = 0.5$ is shown in Supplementary Fig. 12 with the solid lines. The dynamics here represent a simple gradient descent of the Frank-Oseen free energy with respect to the polar angle $\phi = \phi(x,t)$ and the tilt amplitude $\theta_a = \theta_a(x)$. These dynamics are not necessarily representative of the dynamics of the liquid crystal, which would typically involve hydrodynamic effects. Nevertheless, these "model A" [1] dynamics of the angle variables give us a qualitative picture of how the free energy may relax when a tilt is introduced. The time-dependence of the polar angle $\phi = \phi(x,t)$ and tilt amplitude $\theta_a = \theta_a(x)$ are given by

$$\begin{cases} \frac{\partial\phi}{\partial t} = -D_\phi\frac{\delta F}{\delta\phi} \\ \frac{\partial\theta_a}{\partial t} = -D_\theta\frac{\delta F}{\delta\theta_a} \end{cases}, \quad \text{(S4)}$$

where F is the total free energy (Frank-Oseen and anchoring energy), with the ansatz $\theta(x,z) = \theta_a(x)\sin(2\pi z/d)$. We will set the relaxation coefficients to unity $D_\theta = D_\phi = 1$ for simplicity. For our initial condition, we take the planar equilibrium configuration for the polar angle ϕ (black line in Fig. Z), and a nearly constant tilt $\theta_a \approx 0.5$ at the location of the soliton. The boundary conditions on our numerical solutions are $\phi = \pi$ and $\theta_a = 0$. After an initial transient, the tilt localizes at the center of the two π-solitons, as shown in the red line in Supplementary Fig. 12. Then, evolving the dynamics in Eq. (S4) pushes apart the π-solitons, creating a region with $\phi = 0{,}2\pi$, as shown with the solid lines in Supplementary Fig.12. The tilt amplitude θ_a forms two traveling bumps that move along with the π-solitons, as shown with dashed lines in Supplementary Fig. 12.

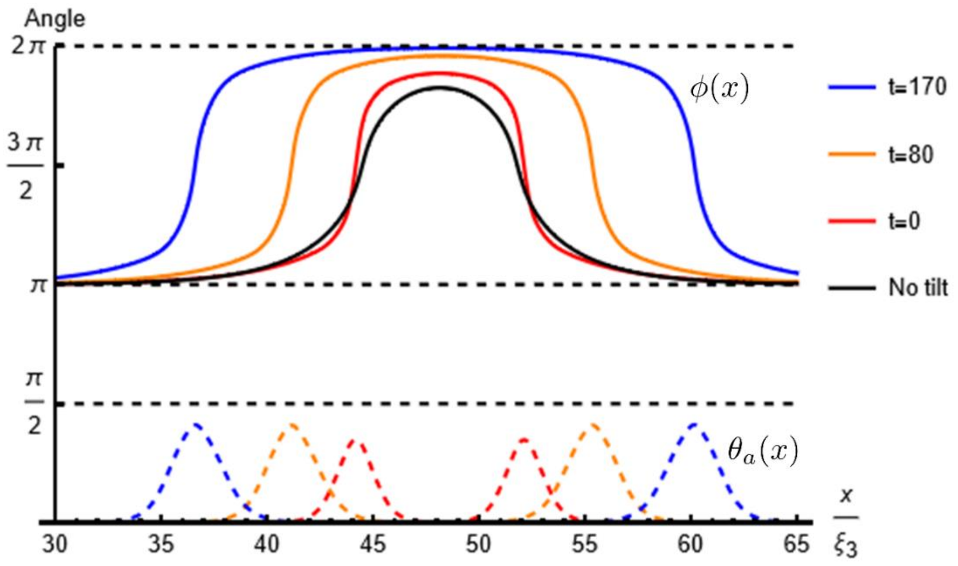

Supplementary Figure 12. Relaxation of a non-topological soliton via tilt. Time-evolution of a non-topological soliton with $\phi = \pi$ as $x \to \pm\infty$ which, in the case of no tilt, is shown with a solid black line (for $\kappa = 10$, $d/\xi_3 = 20$, $\omega = 0.1$, $K_2/K_3 = 0.5$). By introducing a tilt θ, the soliton [after an initial transient which sharpens the domain walls (red line)] splits into two regions with a substantial tilt (dashed lines) where ϕ rotates by π. These two regions spread apart from each other due to the favorable anchoring energy for $\phi = 2\pi$, as shown with the solid lines. We consider here simple relaxation dynamics, Eq. (S4), solved numerically.

VII. Energy of a twisted state.

A cell with a similar alignment of **P** at the two plates carries no elastic energy. When $\varphi(z) = 0$, the energy per unit area is $f = 0$, while for $\varphi(z) = \pi$, it is $f = 4W_P$. Consider a cell in which cooling results in the selection of antiparallel alignment directions, e.g., $\varphi(z = 0) = 0$ and $\varphi(z = d) = \pi$. These boundary conditions produce a twist of $\hat{\mathbf{n}}$ and **P** along the z-axis, Supplementary Fig.13. If there are no in-plane variations of $\varphi(z)$, the free energy per unit area reads

$$f_t = \frac{K_2}{2}\int_0^d \left(\frac{\partial\varphi}{\partial x}\right)^2 dz + \frac{W_Q}{2}[\sin^2\varphi_0 + \sin^2(\varphi_d - \pi)] - W_P[\cos\varphi_0 + \cos(\varphi_d - \pi)] + 2W_P, \quad \text{(S5)}$$

where φ_0 and φ_d are the actual alignment directions at the bottom and top plate, respectively. The elastic energy of the twist makes these directions different from those imposed by the surface potential, i.e., $\varphi_0 > 0$ and $\varphi_d < \pi$. To make the problem tractable, we assume that the deviations from the anchoring "easy" directions are small, so that

$$f_t = \frac{K_2}{2}\int_0^d \left(\frac{\partial\varphi}{\partial z}\right)^2 dz + \frac{1}{2}\left(W_Q + W_P\right)\varphi_0^2 + \frac{1}{2}\left(W_Q - W_P\right)(\varphi_d - \pi)^2 + 2W_P. \quad \text{(S6)}$$

The corresponding Euler-Lagrange equation $\frac{\partial^2\varphi}{\partial z^2} = 0$ leads to the uniform twist along the z-axis: $\varphi(z) = (\varphi_d - \varphi_0)z/d + \varphi_0$, where the constants of integrations φ_0 and φ_d are found from the balance of the elastic and anchoring torques at the plates

$$-\frac{K_2}{d}(\varphi_d - \varphi_0) + \left(W_Q + W_P\right)\varphi_0 = 0 \text{ and } \frac{K_2}{d}(\varphi_d - \varphi_0) + \left(W_Q - W_P\right)(\varphi_d - \pi) = 0 \quad \text{(S7)}$$

as

$$\varphi_0 = \frac{\pi\xi_2(1-\omega)}{d + 2\xi_2 - \omega^2 d}, \quad \text{(S8)}$$

and

$$\varphi_d = \pi - \frac{\pi\xi_2(1+\omega)}{d + 2\xi_2 - \omega^2 d}; \quad \text{(S9)}$$

here $\xi_2 = K_2/W_Q$ is the (apolar) anchoring extrapolation length associated with the twist torques and thus the twist constant. As expected, the elasticity-driven deviations of **P** from the easy direction at the bottom plate, Eq.(S8), are smaller than the deviations from the direction $\varphi = \pi$ at the top plate, Eq.(S9). The stored anchoring and elastic energies of the equilibrium twist configuration are then

$$f_t = 2W_P + \frac{\pi^2}{2}\frac{K_2\left(1-\omega^2\right)}{d(1-\omega^2) + 2\xi_2}. \quad \text{(S10)}$$

Note that f_t might be larger or smaller than the energy $4W_P$ of the state with $\varphi(z) = \pi$, depending on the cell thickness d. The critical thickness below which f_t is larger than $4W_P$ is $d_c \approx \frac{\pi^2 K_2}{8W_P} \approx 3.6$ µm, where we use the estimates $K_2/W_P \approx 3$ µm.

The principal scaling $\propto 1/d$ of the elastic energy in Eq. (S10) facilitates the relaxation of thin cells into the uniform ground state, $\varphi(z) = 0$. For a qualitative illustration, consider the φ_d-dependence of the energy written following its original form, Eq. (S4), with a constant rate of twist $(\varphi_d - \varphi_o)/d$ and with the equilibrium value of φ_0, specified by Eq. (S8):

$$f_t(\varphi_d)/W_Q = \frac{\xi_Q}{2d}(\varphi_d - \varphi_o)^2 + \frac{1}{2}[\sin^2\varphi_0 + \sin^2(\varphi_d - \pi)] - \omega[\cos\varphi_0 + \cos(\varphi_d - \pi)] + 2\omega. \quad \text{(S11)}$$

With the estimates $K_2 \approx 5$ pN, $W_Q \approx 1.3 \times 10^{-5}$ J/m^2, $\xi_2 = \frac{K_2}{W_Q} = 0.4$ µm, and $\omega = 0.1$, the plots $f(\varphi_d)/W_Q$ for various cell thicknesses demonstrate that the barrier separating the local energy minimum at $\varphi_d = \pi$ is clearly visible for very thick cells, $d = 4.7$ µm and 100 µm, but gradually disappears as d decreases below 2 µm, Supplementary Fig. 13b. It means that the elastic torque helps the thin cells to relax into the ground state with $\varphi(z) = 0$, thus supporting the experimental observation of a monodomain texture at small d.

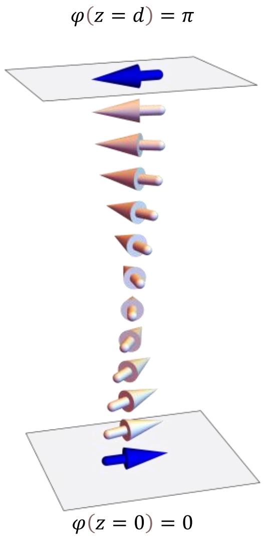

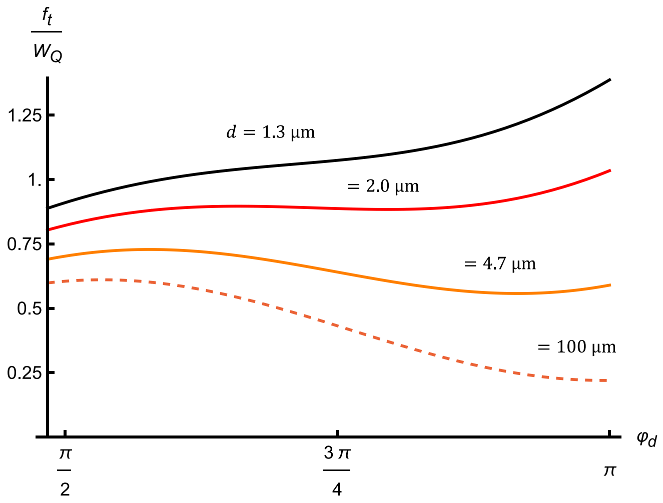

Supplementary Figure 13. Twisted N_F states in planar cells. a, Surface anchoring sets two opposite orientations of the polarization $\boldsymbol{P}$; **b**, the energy of the twisted state in cells of

different thickness d calculated as a function of the azimuthal angle φ_d at the top plate, using Eq.(S11) and parameters specified in the text.

SUPPLEMENTARY REFERENCES

[1] Hohenberg, P. C. & Halperin, B. I. Theory of dynamic critical phenomena. *Rev Mod Phys* **49**, 435-479 (1977)
