# Supplementary material for: Soliton walls paired by polar surface interactions in a ferroelectric nematic liquid crystal
Source: Nat Commun. 2022 Jul 7;13:3932. doi: 10.1038/s41467-022-31593-w (PMC9262936; doi:10.1038/s41467-022-31593-w)
Supplement: Supplementary file 3 — Reporting Summary [file 41467_2022_31593_MOESM3_ESM.pdf]

nature portfolio

Corresponding author(s): Lavrentovich

Last updated by author(s): Jun 13, 2022

Reporting Summary

Nature Portfolio wishes to improve the reproducibility of the work that we publish. This form provides structure for consistency and transparency in reporting. For further information on Nature Portfolio policies, see our Editorial Policies and the Editorial Policy Checklist.

Statistics

For all statistical analyses, confirm that the following items are present in the figure legend, table legend, main text, or Methods section.

n/a	Confirmed	
☐	☒	The exact sample size (*n*) for each experimental group/condition, given as a discrete number and unit of measurement
☐	☒	A statement on whether measurements were taken from distinct samples or whether the same sample was measured repeatedly
☒	☐	The statistical test(s) used AND whether they are one- or two-sided<br>*Only common tests should be described solely by name; describe more complex techniques in the Methods section.*
☒	☐	A description of all covariates tested
☐	☒	A description of any assumptions or corrections, such as tests of normality and adjustment for multiple comparisons
☐	☒	A full description of the statistical parameters including central tendency (e.g. means) or other basic estimates (e.g. regression coefficient) AND variation (e.g. standard deviation) or associated estimates of uncertainty (e.g. confidence intervals)
☒	☐	For null hypothesis testing, the test statistic (e.g. *F*, *t*, *r*) with confidence intervals, effect sizes, degrees of freedom and *P* value noted<br>*Give P values as exact values whenever suitable.*
☒	☐	For Bayesian analysis, information on the choice of priors and Markov chain Monte Carlo settings
☐	☒	For hierarchical and complex designs, identification of the appropriate level for tests and full reporting of outcomes
☒	☐	Estimates of effect sizes (e.g. Cohen's *d*, Pearson's *r*), indicating how they were calculated

Our web collection on statistics for biologists contains articles on many of the points above.

Software and code

Policy information about availability of computer code

Data collection	Data is collected by recording microscopy images using the commercial camera software (QImage). Commercial Hinds MicroImager software is used for Microimager measurement.
Data analysis	The analysis of the images are done using the open source software ImageJ. The calculations and the plots are done using custom written code in Mathematica 12.0.

For manuscripts utilizing custom algorithms or software that are central to the research but not yet described in published literature, software must be made available to editors and reviewers. We strongly encourage code deposition in a community repository (e.g. GitHub). See the Nature Portfolio guidelines for submitting code & software for further information.

Data

Policy information about availability of data

All manuscripts must include a data availability statement. This statement should provide the following information, where applicable:

- Accession codes, unique identifiers, or web links for publicly available datasets
- A description of any restrictions on data availability
- For clinical datasets or third party data, please ensure that the statement adheres to our policy

All data that support the plots within this paper and other findings of this study are available from the corresponding author upon reasonable request.

Human research participants

Policy information about studies involving human research participants and Sex and Gender in Research.

Reporting on sex and gender	n/a
Population characteristics	n/a
Recruitment	n/a
Ethics oversight	n/a

Note that full information on the approval of the study protocol must also be provided in the manuscript.

Field-specific reporting

Please select the one below that is the best fit for your research. If you are not sure, read the appropriate sections before making your selection.

[x] Life sciences [ ] Behavioural & social sciences [ ] Ecological, evolutionary & environmental sciences

For a reference copy of the document with all sections, see nature.com/documents/nr-reporting-summary-flat.pdf

Life sciences study design

All studies must disclose on these points even when the disclosure is negative.

Sample size	The sample size in this study is the thickness of the liquid crystal slab and it is determined by measuring the gap between two bounding glass plates using interferometry techniques. Two different sample thicknesses are considered. Samples of 1-2 um thick and samples of 4-16 um thick. The thinner set of samples shows the polarization monodomain while the thicker set shows polydomain. These sample sizes are sufficient because they cover both possible scenarios of mono- and poly-domain formation.
Data exclusions	The data is only collected in temperature range of 45-55 C where the material is in ferroelectric nemaics phase and far from phase transition temperatures to avoid any memory effect from other phases.
Replication	The results are reproducible, as our error bars are calculated from data of new experiments.
Randomization	Different sets of samples were prepared by different co-authors through the same procedure. The results are reproducible.
Blinding	Two leading co-authors, Bijaya Basnet and Mojtaba Rajabi, performed observations one at a time, i.e. some samples were observed by Bijaya without Mojtaba, the other samples were observed by Mojtaba without Bijaya thus we took blinding precautions and our observations are not biased.

Reporting for specific materials, systems and methods

We require information from authors about some types of materials, experimental systems and methods used in many studies. Here, indicate whether each material, system or method listed is relevant to your study. If you are not sure if a list item applies to your research, read the appropriate section before selecting a response.

Materials & experimental systems

n/a	Involved in the study
[x]	[ ] Antibodies
[x]	[ ] Eukaryotic cell lines
[x]	[ ] Palaeontology and archaeology
[x]	[ ] Animals and other organisms
[x]	[ ] Clinical data
[x]	[ ] Dual use research of concern

Methods

n/a	Involved in the study
[x]	[ ] ChIP-seq
[x]	[ ] Flow cytometry
[x]	[ ] MRI-based neuroimaging
